# A streamlined guide RNA screening system for genome editing in *Sorghum bicolor*

**DOI:** 10.1186/s13007-023-01058-2

**Published:** 2023-08-26

**Authors:** Jeong Sun Lee, Su-Ji Bae, Jin-Soo Kim, Changsoo Kim, Beum-Chang Kang

**Affiliations:** 1 Center for Genome Engineering, Institute for Basic Science, Daejeon, Republic of Korea; 2 Department of Crop Science, College of Agricultural and Life Sciences, Chungnam National University, Daejeon, Republic of Korea; 3 National Research Safety Headquarters, Korea Research Institute of Bioscience and Biotechnology, Cheongju, Republic of Korea; 4 NUS Synthetic Biology for Clinical & Technological Innovation (SynCTI) and Department of Biochemistry, National University of Singapore, Singapore, Singapore; 5 Department of Horticulture, College of Agricultural Life Science, Jeonbuk National University, Jeonju, Republic of Korea

**Keywords:** Sorghum genome editing, Protoplast isolation, *Sorghum bicolor*, CRISPR/Cas9, DNA-free genome editing

## Abstract

**Background:**

Genome editing tools derived from clustered regularly interspaced short palindromic repeats (CRISPR) systems have been developed for generating targeted mutations in plants. Although these tools hold promise for rapid crop improvement, target-specific guide RNAs exhibit variable activity. To improve genome editing, a rapid and precise method for evaluating their efficiency is necessary.

**Results:**

Here we report an efficient system for screening single guide RNAs (sgRNAs) for genome editing in sorghum using a transient protoplast transfection assay. Protoplasts were isolated from leaves from sorghum plants cultivated under three different conditions. Cultivation for three days of continuous darkness following seven days with a 16-h light and 8-h dark photoperiod resulted in the highest yield of viable protoplasts and the highest protoplast transfection efficiency. We tested both plasmid-mediated and ribonucleoprotein-based delivery to protoplasts, via polyethylene glycol-mediated transfection, of CRISPR components targeting the sorghum genome. The frequencies of small insertions and deletions induced by a set of sgRNAs targeting four endogenous sorghum genes were analyzed via targeted deep sequencing. Our screening system induced indels in sorghum protoplasts at frequencies of up to 77.8% (plasmid) and 18.5% (RNP). The entire screening system was completed within 16 days.

**Conclusions:**

The screening system optimized in this study for predicting sgRNA activity for genome editing in sorghum is efficient and straightforward. This system will reduce the time and effort needed for sorghum genome editing.

**Supplementary Information:**

The online version contains supplementary material available at 10.1186/s13007-023-01058-2.

## Background

Programmable genome editing tools derived from clustered regularly interspaced short palindromic repeats (CRISPR) systems have been used to edit a variety of crop genomes [1]. Introduction of gene modifications, such as insertions, deletions, and substitutions, enables rapid trait modification and analysis of gene function. CRISPR/CRISPR-associated protein 9 (Cas9) [2–5] and CRISPR/CRISPR-associated endonuclease in Prevotella and Francisella 1 (Cas12a, Cpf1) [6, 7] typically generate double strand breaks (DSBs) at target regions containing sequences complementary to a single guide RNA (sgRNA). Repair of such breaks by non-homologous end joining often leads to small insertions or deletions (indels), resulting in frameshifts and gene knock-out, whereas homology-directed repair, in the presence of donor DNA, can be used for gene replacement [8]. Base editing systems, which involve fusions of Cas9 nickase and a deaminase, induce precise nucleotide substitutions in the target region without generating DSBs. Cytosine base editor (CBE) [9–12] generates C∙G to T∙A conversions in the target site window whereas adenosine base editor (ABE) [13–15] generates A∙T to G∙C conversions. The recently developed prime editors [16, 17], which consist of a Cas9 nickase-reverse transcriptase fusion protein and a prime editing guide RNA, enable the targeted generation of insertions, deletions, and point mutations in plant genomes without donor DNA and without producing DSBs. All of these CRISPR-derived genome editing systems can be used to enhance crop breeding.

Sorghum is an important crop in global agriculture. Grown as a grain, forage, and bioenergy crop, sorghum is the fifth most widely grown cereal crop; its use is especially extensive in semi-arid regions due to its heat and drought tolerance [18, 19]. Additionally, its small genome size (~ 730 Mb) makes sorghum an attractive model for C4 cereal crop functional genomics [20, 21]. Despite these merits, genome editing in sorghum has fallen behind that of other cereals because of the difficulty of obtaining appropriate material, such as immature embryos, constraining sorghum tissue culture and stable transformation. Previous reports of CRISPR/Cas9-mediated genome editing in sorghum have described targeting the centromere-specific histone 3 (*SbCENH3*) gene [22], the α-kafirin gene family [23], the flowering T locus (*SbFT*), and the gibberellin 2-beta-dioxygenase 5 (*SbGA2ox5*) gene [24]. Both *Agrobacterium*-mediated [22] and microprojectile bombardment-derived [25] transformation methods have been developed for sorghum, but research that aims to increase the efficiency of sorghum genome editing, with sgRNA screening as a critical factor to minimize time and effort, is still lacking.

Transient protoplast transfection assays are a versatile, rapid, and high-throughput method for investigating gene expression [26] and subcellular localization of proteins [27] as well as for assessing genome editing efficiency in plants. Reliable protocols involving polyethylene glycol (PEG)-mediated protoplast transfection have been established for various species, such as *Arabidopsis thaliana* [26], rice (*Oryza sativa*) [28], and maize (*Zea mays*) [29]. Protoplast transfection with genome editing tools has been successfully performed, promising highly efficient genome editing not only with Cas9- or Cas12a-mediated systems but also with base editors and prime editors, in lettuce (*Lactuca sativa*) [30], soybean (*Glycine max*) [7], petunia (*Petunia x hybirda*) [31], rapeseed (*Brassica napus*) [14], and rice [17]. Although sorghum protoplast isolation and transfection have previously been used for plasmid-mediated genome editing [32], here we optimized methods for assessing the editing efficiency and off-target effects of specific sgRNAs for sorghum genome editing with both plasmid-mediated and ribonucleoprotein-based protoplast delivery systems, a necessary step for genetic studies and plant biotechnology.

To improve the precision of genome editing and reduce off-target effects, DNA-free genome editing, in which preassembled Cas9-sgRNA ribonucleoproteins (RNPs) are delivered into protoplasts, has been developed. RNPs can cleave the target region immediately without transcription and translation and are then rapidly degraded, so off-target effects are reduced compared those associated with plasmid-mediated delivery of CRISPR components. RNP systems have been successfully used for genome editing of soybean [7], lettuce [30], petunia [31], wheat (*Triticum aestivum*) [33], and pepper (*Capsicum annuum*) [34]. However, RNP-mediated genome editing in sorghum has not yet been reported.

Here, we screened sgRNAs for targeted mutagenesis of four endogenous sorghum genes that are involved with flowering time (*FT* genes) and vegetative branching (*TIL1* gene): *SbFT1* (Sb10g003940), *SbFT8* (Sb03g034580), *SbFT12* (Sb06g012260), and *SbTIL1* (Sb06g019010) [35–37].

In this study, we present a screening system for precise and highly efficient genome editing in sorghum. We first isolated protoplasts from leaves from sorghum grown under three different cultivation conditions. We analyzed protoplast yield, viability, and transfection efficiency to establish optimal conditions for sgRNA screening. We transfected Cas9-sgRNA expression plasmids into sorghum protoplasts and analyzed the resulting editing efficiencies including that in potential off-target regions by targeted deep sequencing. Furthermore, we tested an RNP system in sorghum protoplasts. Our sgRNA screening system will be a key method for evaluating the activity of sgRNAs for sorghum genome editing.

## Methods

### Plant material

Commercial grain sorghum (*Sorghum bicolor* L. cv. Imky1ho) was used in all experiments. Seeds were sown on commercial bed soil. Seedlings were cultivated under three different conditions (Condition 1: 10 days of 16 h light/8 h darkness; Condition 2: 7 days of 16 h light/8 h darkness and 3 days of 24 h darkness; Condition 3: 3 days of 16 h light/8 h darkness and 7 days of 24 h darkness) at 25 ℃.

### Protoplast isolation

**Fig. 1:**
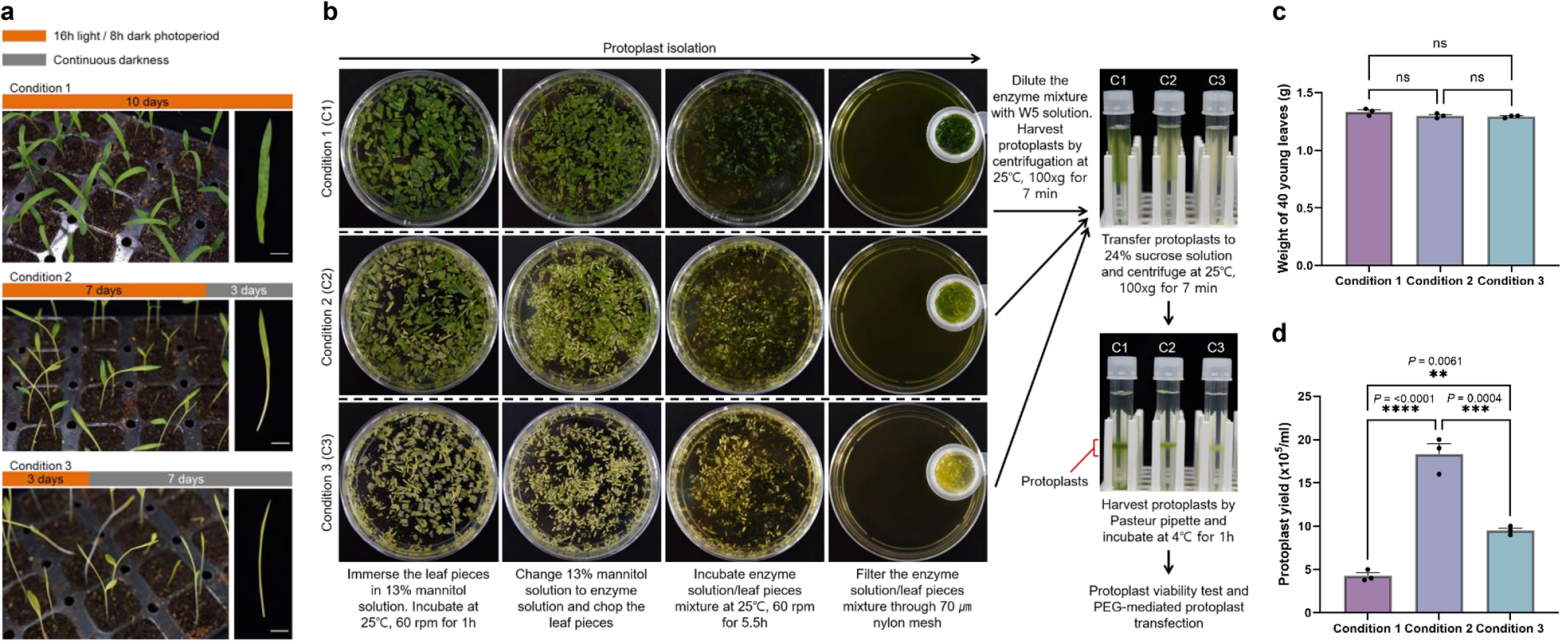
Protoplast isolation from leaves from sorghum plants cultivated under three different conditions. **a** Ten-day-old seedlings grown under different conditions. Condition 1: 16 h light/8 h dark for 10 days; Condition 2: 16 h light/8 h dark for 7 days and continuous darkness for 3 days; Condition 3: 16 h light/8 h dark for 3 days and continuous darkness for 7 days. Scale bars = 1 cm. **b** Workflow of the protoplast isolation procedure. Enzyme-treated protoplasts were harvested by sucrose gradient centrifugation. **c** Weight of leaves, obtained following different cultivation conditions, used for protoplast isolation. **d** Yield of isolated protoplasts from leaves of plants cultivated under each condition. **c** and **d** Values (mean ± s.e.m.) were obtained from three independent experiments. One-way ANOVA analysis was applied. ****P < 0.0001; ***P < 0.001; **P < 0.01; ns, not significant (*P* > 0.05)

Protoplasts were isolated using a protocol described previously [38, 39] with the following modifications: 40 young leaves (Fig. 1a) from plants cultivated under each condition described above were cut into 1 cm long pieces, immersed in a 13% mannitol solution, and incubated at 25 ℃ on a shaker with gentle agitation (60 rpm) for 1 h in the dark, after which the solution was exchanged for enzyme solution (Table 1). Using a razor blade, samples were chopped into pieces about 3–4 mm on a side and incubated at 25 ℃ with 60 rpm agitation for 5.5 h in the dark. The digested mixture was filtered through a 70 μm nylon cell strainer and washed with an equal volume of W5 solution (Table 1). The protoplasts were isolated on a sucrose gradient (24%) by swing-out centrifugation at 100 ×*g* for 7 min. The intact protoplasts were harvested using a Pasteur pipette, after which they were incubated in W5 solution for 1 h at 4 ℃ before being used for the protoplast viability test or protoplast transfection.

**Table 1:** Composition of solutions used for sorghum protoplast isolation and transfection

Solution name	Composition
13 M mannitol	13% mannitol (Duchefa Biochemi, Netherlands)
Enzyme	10 g/L Cellulase (Duchefa Biochemi), 1 g/L Pectolyase (Duchefa Biochemi), 0.97 g/L MES (Sigma-Aldrich, USA), 90 g/L mannitol, CPW salts (27.2 mg/L KH_2_PO_4_, 101 mg/L KNO_3_, 246 mg/L MgSO_4_∙7H_2_O, 0.16 mg/L KI, 0.025 mg/L CuSO_4_∙5H_2_O, 1480 mg/L CaCl_2_∙5H_2_O), pH 5.8
24% sucrose	24% sucrose (Duchefa Biochemi)
W5	154 mM NaCl, 125 mM CaCl_2_∙H_2_0, 5 mM KCl, 5 mM glucose, 1.5 mM MES, pH 5.8
MMG	4 mM MES, 0.4 M mannitol, 15 mM MgCl_2_, pH 5.7
WI	4 mM MES, 0.5 M mannitol, 20 mM KCl, pH 5.7
PEG-CaCl_2_	40% (wt/vol) PEG4000 (Sigma-Aldrich), 0.2 M mannitol, 0.1 M CaCl_2_∙H_2_O

### Protoplast viability test

Evans blue dye solution (0.02%, Sigma-Aldrich) was mixed with an equal volume of sorghum protoplasts in W5 solution and the mixture was incubated at 25 ℃ for 10 min. The numbers of live (unstained) and dead (stained) protoplasts were determined on a hemocytometer under a light microscope. Protoplast viability was calculated as the number of unstained protoplasts / total number of protoplasts.

### Guide RNA design

We compared the nucleotide sequences of the target genes with the corresponding reference sequences [*SbFT1* (Sb10g003940), *SbFT8* (Sb03g034580), *SbFT12* (Sb06g012260), and *SbTIL1* (Sb06g019010)] using Sanger sequencing (capillary electrophoresis sequencing, Macrogen, Korea) of PCR amplicons (Additional files 1 and 2). Guide RNAs were designed from the analyzed sequences using Cas-Designer [40] with the Sorghum genome database (v1.0). We selected guide RNAs with high microhomology-associated out-of-frame scores with few potential off-targets effects using Cas-offinder [41] with Sorghum genome database (v1.0). Nucleotide alignments were performed using Geneious (version 8.1.9).

### Plasmid construction

To construct the pJ4 plasmid (used for sgRNA and Cas9 expression), we changed the initial base of the sgRNA expression module sequence in pBUN421 (Addgene No. 62204) [42] from guanine to adenine, because the U3 promoter requires an adenine at the transcription start site, using Gibson assembly [43]. pJ4 uses the maize ubiquitin (ZmUbi-1) promoter and Nos terminator to express *Zea mays* codon-optimized Cas9. In preparation for generating sgRNA-expressing plasmids, pairs of oligonucleotides representing the desired sgRNA sequences were synthesized by MOPC (macrogen oligonucleotide purification cartridge, Macrogen, Korea) (Additional file 1). Next, an annealing reaction mixture (1 × T4 ligase buffer containing 25 μM of each of the two oligonucleotides) was incubated at 95 ℃ for 3 min, then cooled gradually (0.1 ℃/s) to 25 ℃) to allow annealing. Plasmids expressing sgRNAs were constructed by T4 ligation (New England Biolabs) of annealed oligonucleotides into BsaI-digested pJ4 vector at 25 ℃ for 20 min (Additional file 3). All plasmids used in the transient protoplast transfection assay were purified using Plasmid Plus Maxiprep kits (QIAGEN).

### In vitro transcription of sgRNA

DNA templates for sgRNA transcription were prepared by oligonucleotide extension (Additional file 1) using Phusion High-Fidelity DNA polymerase. sgRNAs were synthesized via runoff reactions using T7 RNA polymerase (New England BioLabs) according to the manufacturer’s protocol. In brief, a reaction mixture (1.5 μg of DNA template, 4 mM each of ATP, CTP, GTP, and UTP, 14 mM MgCl_2_, 10 mM DTT, 1 × T7 polymerase buffer, 500 units of RNase inhibitor, and 3750 units of T7 polymerase) was incubated overnight at 37 ℃, after which the synthetic sgRNAs were purified using a PCR purification kit (GeneAll).

### In vitro cleavage assay

Genomic DNA was isolated from sorghum leaves using a DNeasy Plant Mini Kit (QIAGEN). Regions spanning the target sites were amplified from the genomic DNA using target-specific primer sets (Additional file 1). Templates (120 ng) were incubated in 1 × NEB buffer 3.1 at 37 ℃ for 2 h with Cas9 protein (2 μg) and sgRNA (1.5 μg). RNase A (4 μg) was then added to the reaction mixture, which was incubated at 37 ℃ for 30 min to remove the sgRNA. The products were then purified using a PCR purification kit (Geneall) and analyzed by 2% agarose gel electrophoresis.

### Protoplast transfection

Sorghum protoplasts (5 × 10^4^) in MMG solution (Table 1) were transfected with the Cas9-sgRNA expression plasmid (20 μg) or preassembled RNPs [Cas9 protein (30 μg) and in vitro transcribed sgRNA (80 μg)] by PEG-mediated transfection. Cas9 and the in vitro transcribed sgRNA were premixed at 25 ℃ for 20 min to make the RNPs. The PEG-protoplast mixture was incubated at 25 ℃ for 20 min and washed 3 times with an equal volume of W5 solution (Table 1) with gentle inversion. Protoplasts were pelleted by swing-out centrifugation at 100 ×*g* for 5 min and then resuspended in WI solution. Transfected protoplasts were incubated at 25 ℃ for 72 h in the dark. To measure transfection efficiency, protoplasts were transfected with a plasmid expressing enhanced green fluorescent protein (GFP) and incubated at 25 ℃ for 36 h in the dark. GFP fluorescence was measured using Zeiss confocal microscopy (LSM 800, GFP: 400–650 nm).

### Targeted deep sequencing

Genomic DNA was extracted from protoplasts that had been transfected with the Cas9-sgRNA-expression plasmid or RNPs using a DNeasy Plant Mini Kit (QIAGEN). The target region and potential off-target sites were amplified from genomic DNA with paralogue-specific primer sets (Additional file 1). Multiplexing indices and sequencing adaptors were added to the amplicons by additional rounds of PCR. High-throughput sequencing was performed using Illumina Miniseq with equal amounts of the DNA libraries. The paired-end sequencing files were analyzed by Cas-analyzer [44], available at the RGEN tools site (www.rgenome.net).

### Statistical analysis

All experiments were conducted with three independent replicates. Statistical analysis of the numerical data was performed using GraphPad Prism (9.5.1). For multiple pairwise comparisons, the data were presented as mean ± standard error of the mean (s.e.m), and compared using a one-way ANOVA analysis followed by Tukey’s multiple comparisons test.

## Results

### Development of a cultivation protocol to increase protoplast yield from sorghum leaves

To establish a procedure for efficient protoplast isolation, sorghum plants were cultivated under three different conditions to assess if darkness affects protoplast yield [38]. We isolated protoplasts from young leaves from sorghum plants cultivated under the following conditions: Condition 1: 10 days of 16 h light/8 h darkness; Condition 2: 7 days of 16 h light/8 h darkness and 3 days of 24 h darkness; Condition 3: 3 days of 16 h light/8 h darkness and 7 days of 24 h darkness (Fig. 1a). We isolated protoplasts from 40 young leaves from plants grown under each condition; our workflow is portrayed in Fig. 1b. We observed that the amount of chloroplasts in leaves decreased as the time in darkness increased. Furthermore, we found that although the weights of leaf samples did not significantly differ between cultivation conditions (Fig. 1c), the protoplast yield did differ (Fig. 1d). The efficiency of protoplast isolation was the highest (up to 1.6 × 10^6^/mL) from leaves cultivated under Condition 2. These results show that a period of cultivation in darkness improves protoplast yield from sorghum.

### Determination of protoplast viability and transfection efficiency

**Fig. 2:**
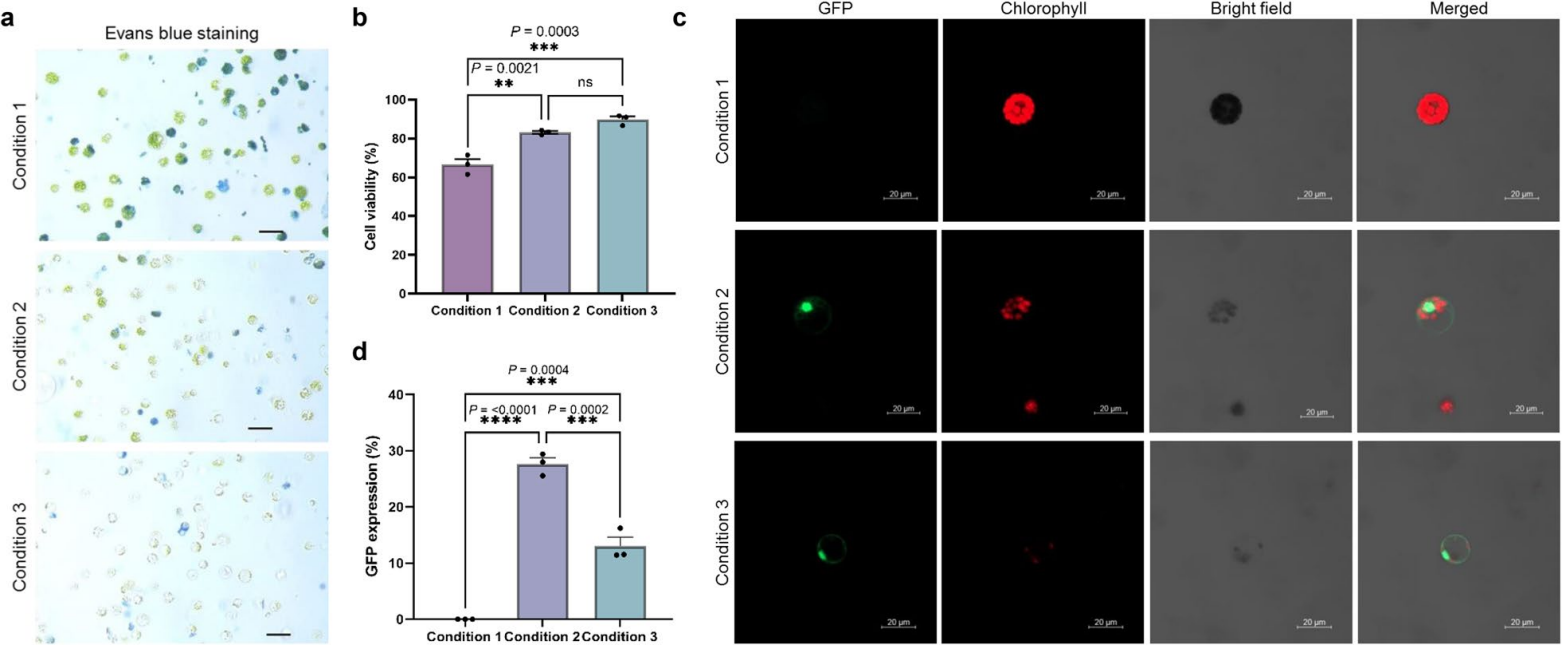
Viability and transfection efficiency of protoplasts from plants cultivated under three different conditions. **a** Evans blue staining of protoplasts isolated from plants cultured under each condition. Dead cells and debris are stained blue. Scale bars = 50 µm. **b** The viability of isolated protoplasts was measured by determining the percentage of protoplasts that were not stained with Evans blue solution, using a hemacytometer. Viabilities (mean ± s.e.m.) were calculated from n = 3 independent experiments. **c** GFP expression in protoplasts that had been isolated from plants cultivated under three different conditions and then transfected with a plasmid expressing GFP fused to a nuclear localization signal. Scale bars = 20 µm. **d** Transfection efficiencies (mean ± s.e.m.) were measured as the percentage of protoplasts expressing GFP. Efficiencies were obtained from n = 3 independent replicates. **b** and **d** Values (mean ± s.e.m.) were obtained from three independent experiments. One-way ANOVA analysis was applied. ****P < 0.0001; ***P < 0.001; **P < 0.01; ns, not significant (*P* > 0.05)

To investigate whether our protoplast isolation method was applicable for screening sgRNA activity, we measured protoplast viability and transfection efficiency. Protoplast viability was determined using Evans blue solution, which stains dead but not living protoplasts (Fig. 2a). 67%, 84%, and 89% of protoplasts from plants cultivated under Conditions 1, 2, and 3, respectively, were found to be intact and healthy (Fig. 2b). To examine the transfection efficiency for each class of protoplast, we delivered a GFP expression plasmid by PEG-mediated transfection (Fig. 2c). The frequency of protoplasts expressing GFP was highest (up to 29%) when protoplasts were isolated from plants cultured under Condition 2 (Fig. 2d). Protoplasts isolated from plants grown under Condition 1, however, exhibited no GFP signal. Taken together, these results show that a period of cultivation in continuous darkness increases both protoplast yield and transfection efficiency. In further experiments, we used protoplasts isolated from plants cultured under Condition 2.

### Screening of sgRNA activity in sorghum protoplasts

**Fig. 3:**
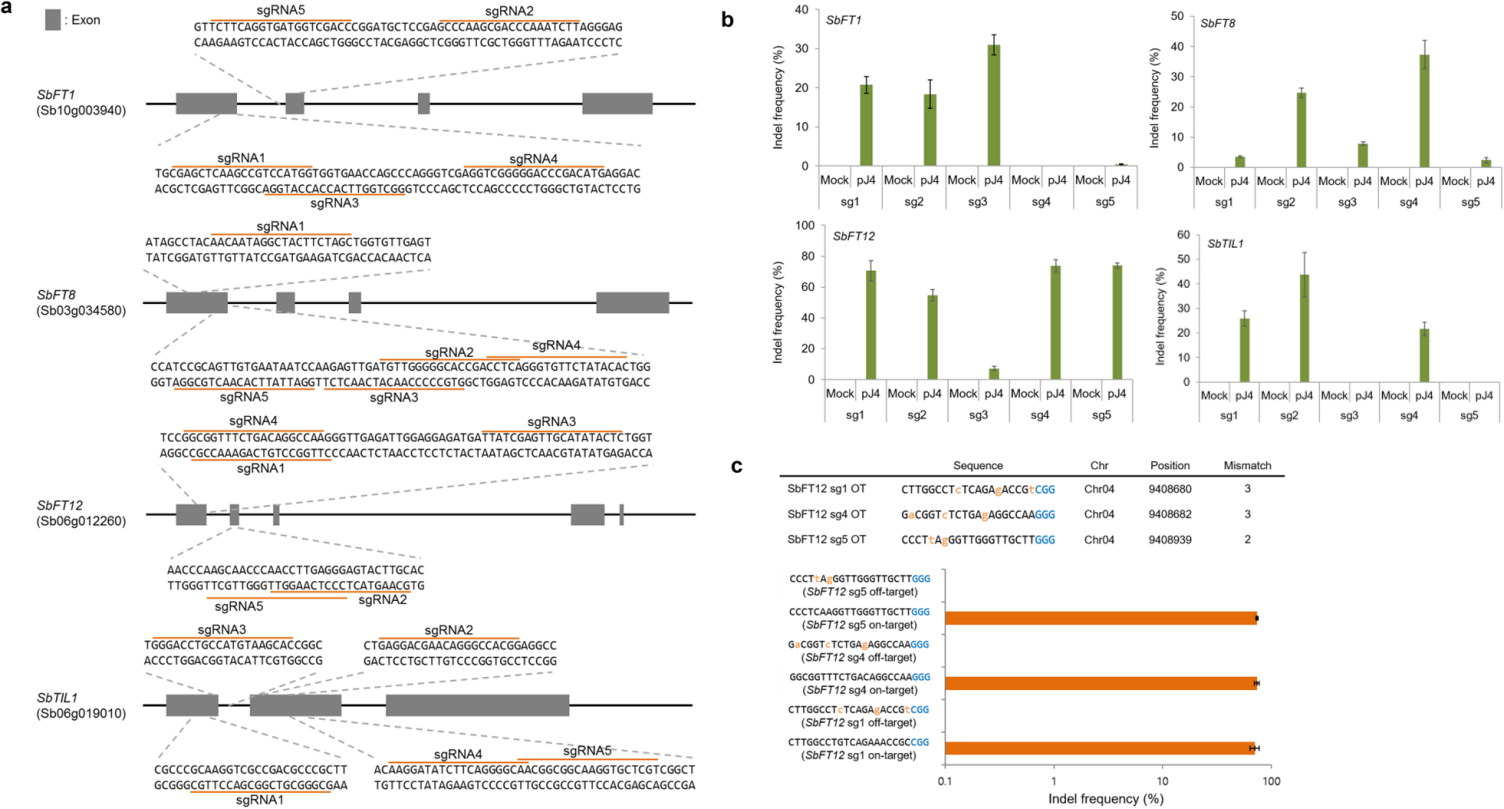
Plasmid-mediated genome editing in sorghum. **a** Target sequences in *SbFT1*, *SbFT8*, *SbFT12*, and *SbTIL1*. **b** Indel frequencies induced by each sgRNA 72 h after transient protoplast transfection. **c** Evaluation of genome editing at candidate off-target sites in transfected protoplasts. PAM sequences and mismatched nucleotides are shown in blue and orange, respectively. **b** and **c **Indel frequencies (mean ± s.e.m.) were obtained from n = 3 independent experiments

Encouraged by these results, we investigated whether the CRISPR-Cas9 system could efficiently edit the sorghum genome. We designed five sgRNAs for each of four genes (*SbFT1*, *SbFT8*, *SbFT12*, and *SbTIL1*) in silico and constructed plasmids expressing each of the sgRNAs by Golden Gate cloning (Fig. 3a and Additional file 1). The Cas9-sgRNA-encoding plasmids were transfected into protoplasts via PEG-mediated delivery and the indel frequency at each target was analyzed by targeted deep sequencing at three days after transfection. The method led to efficient genome editing, with indel frequencies of up to 32.7% for *SbFT1*, 41.8% for *SbFT8*, 77.8% for *SbFT12*, and 54.1% for *SbTIL1* (Fig. 3b). Indels were observed at 85% (17/20) of the target sites, indicating that 17 of the 20 tested sgRNAs exhibited activity. We observed that most editing patterns included either a single nucleotide insertion or a 1–2 nucleotide deletion at the position 3 base pairs upstream of the protospacer adjacent motif (PAM) sequence (Additional file 4).

### Analysis of off-target effects

To evaluate sgRNA specificity, we chose three highly active sgRNAs targeted to the *SbFT12* gene (sg1, sg4, and sg5), which had exhibited editing efficiencies ranging from 63.2% to 77.8% at the target sites. We used the algorithm Cas-OFFinder to identify potential off-target sites, which could contain up to three nucleotide mismatches relative to the target sites, in the sorghum genome. Following protoplast transfection with the Cas9-sgRNA-encoding plasmids, we amplified the potential off-target sites from the protoplast genomic DNA using target-specific primers and sought to identify any off-target effects by targeted deep sequencing. No indels were observed at any of the potential off-target regions (Fig. 3c). These results suggest that plasmid-mediated delivery of Cas9 and sgRNA can be utilized for robust, precise editing in sorghum protoplasts.

### DNA-free genome editing via RNP transfection

**Fig. 4:**
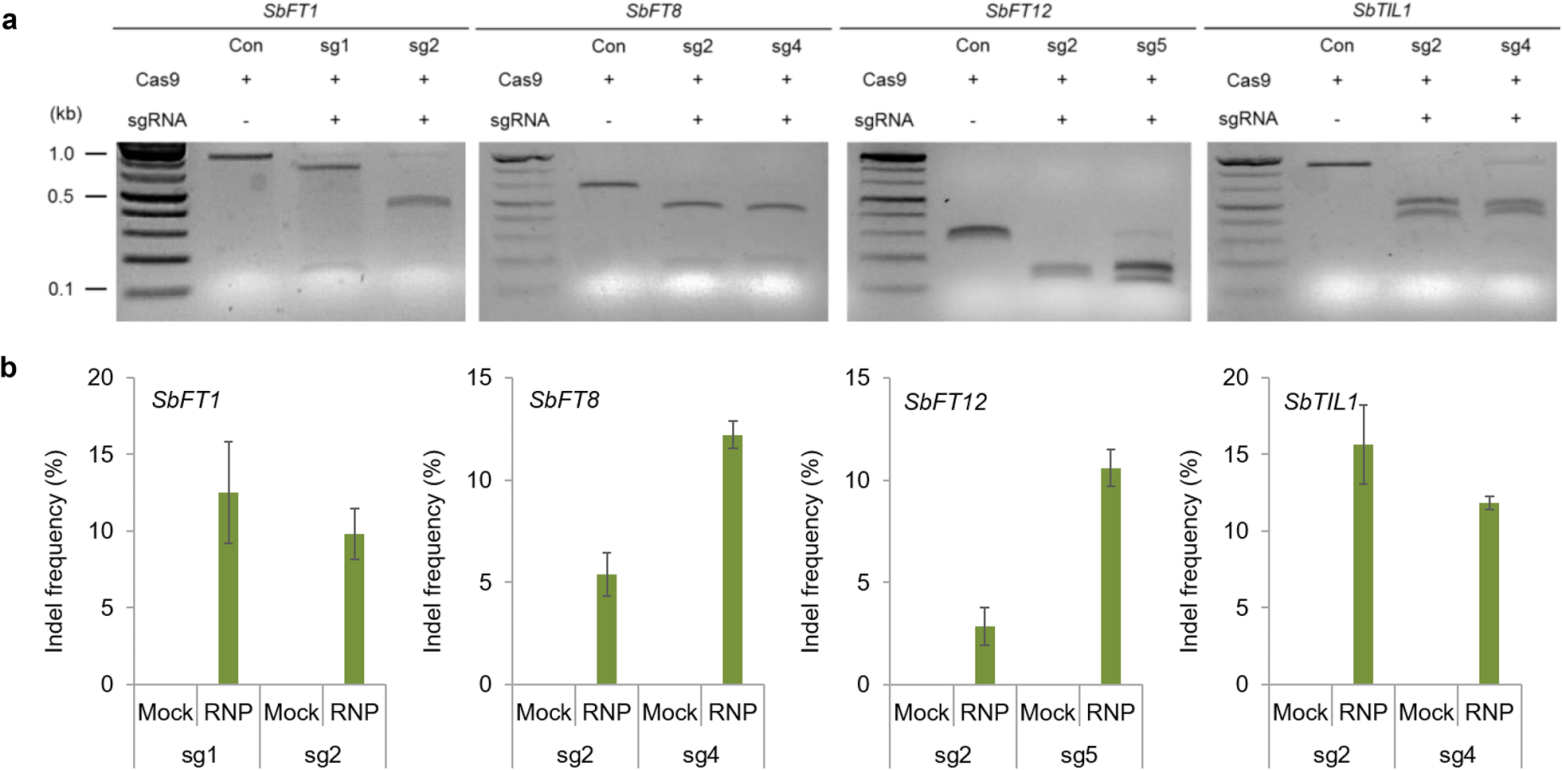
DNA-free genome editing in sorghum. **a** In vitro cleavage assay to examine Cas9-sgRNA RNP activity in the indicated target regions. **b** Indel frequencies at the corresponding endogenous sites induced by preassembled RNPs and determined by targeted deep sequencing. Indel frequencies (mean ± s.e.m.) were obtained from n = 3 independent experiments

To test the efficiency of DNA-free genome editing mediated by Cas9 protein-sgRNA RNPs in sorghum protoplasts, we selected two efficient sgRNAs for each target gene from the experiments described above. First, we used an in vitro cleavage assay to measure the RNP activity in the target regions. We incubated in vitro transcribed sgRNA and recombinant Cas9 protein with a DNA fragment containing the target sequence. Using gel electrophoresis, we confirmed that each RNP complex cleaved the target region (Fig. 4a).sgRNA-Cas9 RNPs with confirmed in vitro activity were transfected into sorghum protoplasts and the indel frequency at the target sites was analyzed three days after transfection by targeted deep sequencing. We observed indel frequencies of up to 14.5% for *SbFT1*, 12.8% for *SbFT8*, 11.6% for *SbFT12*, and 18.5% for *SbTIL1* (Fig. 4b). As seen in the plasmid-mediated genome editing, indel patterns included either a single nucleotide insertion or a 1–2 nucleotide deletion at the position 3 base pairs upstream of the PAM sequence (Additional file 5). These results indicate that the RNPs successfully entered sorghum protoplasts and induced indels in the target regions.

## Discussion

**Fig. 5:**
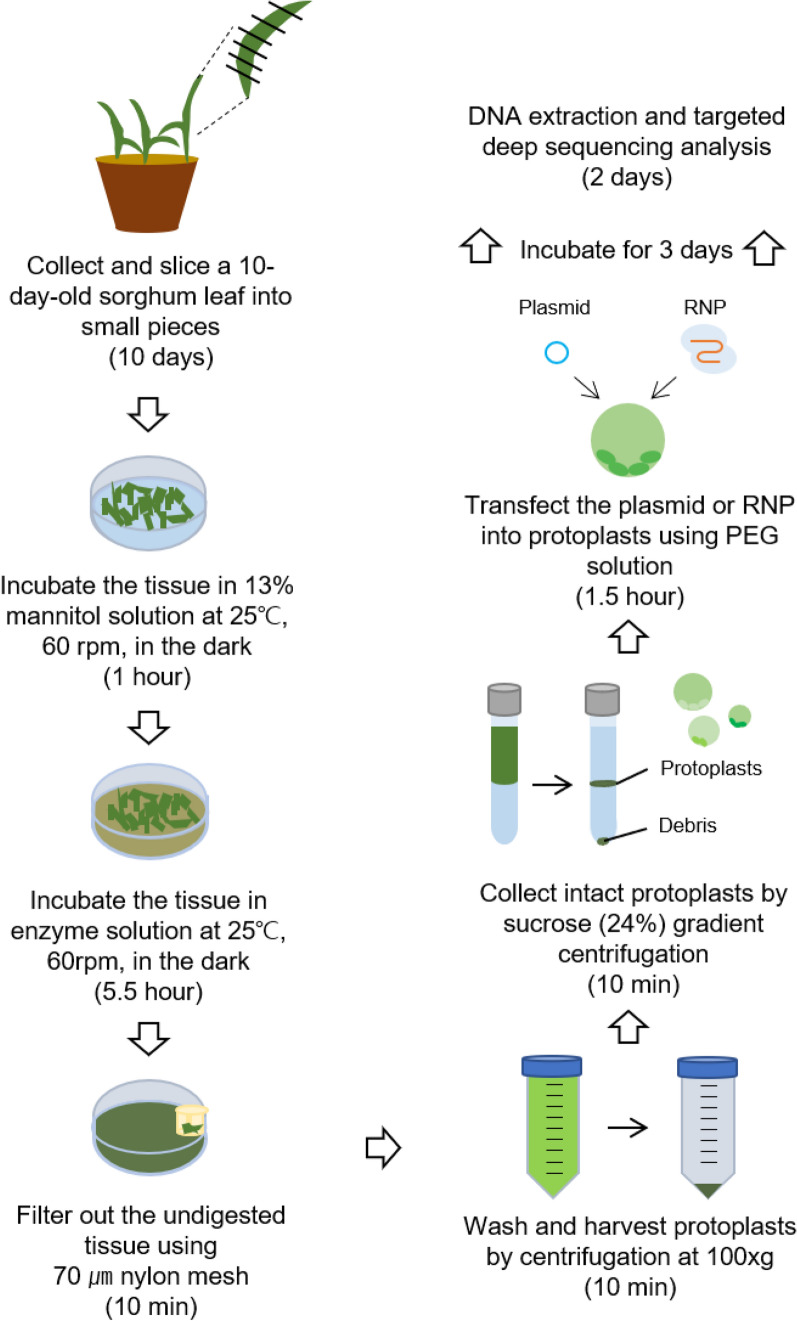
Schematic overview of the sgRNA screening system in sorghum. The time required for each step is indicated in parentheses. Plasmids or preassembled RNPs were delivered into sorghum protoplasts, and editing efficiencies were determined by targeted deep sequencing. The whole process can be completed within 16 days

A few cases of sorghum genome editing have been previously reported [22–24]. Based on former studies, successful plant genome editing requires active sgRNAs and high transformation efficiency. In previous work, sgRNAs designed in silico resulted in editing efficiencies at target genes that differed from the expected result, due to factors such as chromatin accessibility and sequence context. Screening sgRNAs using transient expression in protoplasts is a rapid and stable cell-based method for evaluating their genome editing efficiency. Pre-screening sgRNAs can reduce the number of sgRNAs needed for effectively generating genome-edited plants by transformation. Here, we optimized a sgRNA screening system for precise, efficient sorghum genome editing (Fig. 5). Results from each step of our protocol were verified by multiple tests: determination of protoplast yields from plants grown under three different conditions, protoplast viability and transfection efficiency using Evans blue staining and GFP expression, and the efficiency and precision of editing induced by various sgRNAs by targeted deep sequencing.

We found that a period of cultivation in darkness could enhance the protoplast yield and transfection rate. Condition 2 (7 days of 16 h light/8 h darkness and 3 days of 24 h darkness) resulted in a higher yield of protoplasts (1.83 × 10^6^/mL) and more efficient transfection (29.4%) compared to the other conditions (Condition 1: 10 days of 16 h light/8 h darkness; Condition 3: 3 days of 16 h light/8 h darkness and 7 days of 24 h darkness). Cultivation in darkness improved protoplast yield and transfection, but Condition 2 resulted in 2.2-fold higher transfection efficiency than did Condition 3, which included the longest period of darkness. This observation suggests that the length of time in which plants are cultivated in darkness must be optimized to guarantee the highest yield and transfection efficiency of sorghum protoplasts. We recommend optimizing this variable before conducting genome editing experiments, as cell viability and transfection efficiency differ depending on the genotype and the cultivation conditions.

We showed here that CRISPR/Cas9-based genome editing using sgRNAs designed in silico resulted in indel frequencies of up to 77.8% at the target site. Although each sgRNA was associated with a different editing efficiency as measured by the transient protoplast transfection assay, 85% of these sgRNAs showed editing activity in this study. We also found that active sgRNAs induced indels at similar regions in each target gene (sg1 and sg3 in *SbFT1*; sg2 and sg4 in *SbFT8*; sg1, sg2, sg4, and sg5 in *SbFT12*), suggesting the existence of hot spots (Fig. 3). Too few sgRNAs were studied to make a general rule, but we propose that this observation could serve as a clue for sgRNA design and selection in future experiments. Recently a study reported that use of the endogenous U6 promoter (SbU62.3) in Cas9-sgRNA-encoding plasmids increased genome editing efficiency and the homozygous/bi-allelic editing rate compared to the TaU6 promoter, which is widely used in many crop plants [45]. Our guide RNA screening system could be used with this U6 promoter to improve editing efficiency further.

Our study suggests that DNA-free genome editing could also be a valuable tool for sorghum breeding involving genome editing. We succeeded in editing four different genes (*SbFT1*, *SbFT8*, *SbFT12*, and *SbTIL1*) using Cas9-sgRNA RNPs, observing indel frequencies of up to 18.5%. Relative to plasmid-based delivery systems, RNPs are functional for less time, which can be beneficial for lowering the frequency of off-target effects. Additionally, there is no need to be concerned about transgene integration into the host genome with an RNP system, another advantage when the goal is to improve crop strains. Although the RNP-mediated editing efficiency in this study was lower than that of DNA-mediated genome editing, RNPs could provide an attractive alternative for precise genome editing with a decreased frequency of unintended cleavage sites. The efficiency of protoplast regeneration, an important factor in generating gene-edited plants, is determined mainly by the genotype [46], and it would be very useful to identify the genotype with the highest regeneration efficiency among sorghum cultivars in future studies. An optimized sorghum protoplast regeneration system could be combined with our DNA-free genome editing method to accelerate the generation of transgene-free mutants for practical breeding and the commercial market.

Our sgRNA screening system could also be applied to other genome editing tools. For example, it could be adapted for plant CBE and ABE systems to determine exactly which point mutations would be induced by specific sgRNAs, with the aim of developing agronomic traits in sorghum. Our system could also be used to verify the possibility of base editor-mediated targeted saturation mutagenesis [47] to generate gain-of-function variants. Furthermore, we plan to use our system to train a machine learning algorithm [48, 49] to generate a scoring system that predicts which target sites would be most amenable to editing in the sorghum genome.

In summary, we have developed a rapid and precise sgRNA screening system for efficient genome editing in sorghum, which can be completed within 16 days. We successfully isolated sorghum protoplasts and edited target genes in them. The protoplast isolation and transfection steps can also be used to study topics such as gene expression and protein localization. In addition, we used our system to verify that Cas9-sgRNA RNPs are an effective genome editing tool in sorghum.

## Conclusion

We established an efficient and specific CRISPR/Cas9 screening system for the grain sorghum. This system will allow rapid and precise programmable genome editing in sorghum for crop breeding and plant biotechnology.

### Supplementary Information

**Additional file 1: **Primers used in this study.

**Additional file 2: **Sanger sequencing of relevant regions in target genes. **a** *SbFT1*, **b** *SbFT8*, **c** *SbFT12*, and **d** *SbTIL1*. Single nucleotide polymorphisms are indicated in turquoise.

**Additional file 3: **Insertion of sgRNA-encoding sequences into the pJ4 plasmid. Target sequence annealed oligonucleotides were ligated into the BsaI-digested plasmid to construct vectors that express the desired sgRNAs.

**Additional file 4: **Indel patterns at endogenous sorghum loci induced following plasmid-mediated delivery of CRISPR/Cas9 editing components. **a** *SbFT1*, **b** *SbFT8*, **c** *SbFT12*, and **d** *SbTIL1*. Total reads were obtained by targeted deep sequencing. PAM sequences and inserted or deleted nucleotides are indicated in blue and red, respectively. We tested n = 3 biological replicates.

**Additional file 5: **Indel patterns at endogenous sorghum loci induced following RNP-mediated delivery of CRISPR/Cas9 editing components. **a** *SbFT1*, **b** *SbFT8*, **c** *SbFT12*, and **d** *SbTIL1*. Total reads were obtained by targeted deep sequencing. PAM sequences and inserted or deleted nucleotides are indicated in blue and red, respectively. We tested n=3 biological replicates.

## Data Availability

All data generated or analyzed during this study are included in this published article and its associated additional files.

## Abbreviations

CRISPR: Clustered regularly interspaced short palindromic repeats
Cas9: CRISPR-associated protein 9
Cas12a: CRISPR-associated endonuclease in Prevotella and Francisella 1
DSB: Double strand break
sgRNA: Single guide RNA
CBE: Cytosine base editor
ABE: Adenosine base editor
PEG: Polyethylene glycol
RNP: Ribonucleoprotein
GFP: Green fluorescent protein
PAM: Protospacer adjacent motif
