# Supplementary material for: A streamlined guide RNA screening system for genome editing in Sorghum bicolor
Source: Plant Methods. 2023 Aug 26;19:90. doi: 10.1186/s13007-023-01058-2 (PMC10463630; doi:10.1186/s13007-023-01058-2)
Supplement: Supplementary file 1 — Additional file 1: Primers used in this study. [file 13007_2023_1058_MOESM1_ESM.docx]

**Additional file 1.** Primers used in this study.

| **Primer name** | **Primer sequence (5’-3’)** | **Purpose** |
| --- | --- | --- |
| *SbFT1* sg1 F | AGCACGAGCTCAAGCCGTCCATGG | sgRNA cloning in pJ4 |
| *SbFT1* sg1 R | AAACCCATGGACGGCTTGAGCTCG | sgRNA cloning in pJ4 |
| *SbFT1* sg2 F | AGCAGCCCAAGCGACCCAAATCTT | sgRNA cloning in pJ4 |
| *SbFT1* sg2 R | AAACAAGATTTGGGTCGCTTGGGC | sgRNA cloning in pJ4 |
| *SbFT1* sg3 F | AGCAGGCTGGTTCACCACCATGGA | sgRNA cloning in pJ4 |
| *SbFT1* sg3 R | AAACTCCATGGTGGTGAACCAGCC | sgRNA cloning in pJ4 |
| *SbFT1* sg4 F | AGCAAGGTCGGGGGACCCGACATG | sgRNA cloning in pJ4 |
| *SbFT1* sg4 R | AAACCATGTCGGGTCCCCCGACCT | sgRNA cloning in pJ4 |
| *SbFT1* sg5 F | AGCATCTTCAGGTGATGGTCGACC | sgRNA cloning in pJ4 |
| *SbFT1* sg5 R | AAACGGTCGACCATCACCTGAAGA | sgRNA cloning in pJ4 |
| *SbFT8* sg1 F | AGCAAACAATAGGCTACTTCTAGC | sgRNA cloning in pJ4 |
| *SbFT8* sg1 R | AAACGCTAGAAGTAGCCTATTGTT | sgRNA cloning in pJ4 |
| *SbFT8* sg2 F | AGCATGTTGGGGGCACCGACCTCA | sgRNA cloning in pJ4 |
| *SbFT8* sg2 R | AAACTGAGGTCGGTGCCCCCAACA | sgRNA cloning in pJ4 |
| *SbFT8* sg3 F | AGCAGTGCCCCCAACATCAACTCT | sgRNA cloning in pJ4 |
| *SbFT8* sg3 R | AAACAGAGTTGATGTTGGGGGCAC | sgRNA cloning in pJ4 |
| *SbFT8* sg4 F | AGCACCTCAGGGTGTTCTATACAC | sgRNA cloning in pJ4 |
| *SbFT8* sg4 R | AAACGTGTATAGAACACCCTGAGG | sgRNA cloning in pJ4 |
| *SbFT8* sg5 F | AGCAGGATTATTCACAACTGCGGA | sgRNA cloning in pJ4 |
| *SbFT8* sg5 R | AAACTCCGCAGTTGTGAATAATCC | sgRNA cloning in pJ4 |
| *SbFT12* sg1 F | AGCACTTGGCCTGTCAGAAACCGC | sgRNA cloning in pJ4 |
| *SbFT12* sg1 R | AAACGCGGTTTCTGACAGGCCAAG | sgRNA cloning in pJ4 |
| *SbFT12* sg2 F | AGCAGCAAGTACTCCCTCAAGGTT | sgRNA cloning in pJ4 |
| *SbFT12* sg2 R | AAACAACCTTGAGGGAGTACTTGC | sgRNA cloning in pJ4 |
| *SbFT12* sg3 F | AGCATTATCGAGTTGCATATACTC | sgRNA cloning in pJ4 |
| *SbFT12* sg3 R | AAACGAGTATATGCAACTCGATAA | sgRNA cloning in pJ4 |
| *SbFT12* sg4 F | AGCAGGCGGTTTCTGACAGGCCAA | sgRNA cloning in pJ4 |
| *SbFT12* sg4 R | AAACTTGGCCTGTCAGAAACCGCC | sgRNA cloning in pJ4 |
| *SbFT12* sg5 F | AGCACCCTCAAGGTTGGGTTGCTT | sgRNA cloning in pJ4 |
| *SbFT12* sg5 R | AAACAAGCAACCCAACCTTGAGGG | sgRNA cloning in pJ4 |
| *SbTIL1* sg1 F | AGCAGCGGGCGTCGGCGACCTTGC | sgRNA cloning in pJ4 |
| *SbTIL1* sg1 R | AAACGCAAGGTCGCCGACGCCCGC | sgRNA cloning in pJ4 |
| *SbTIL1* sg2 F | AGCAGAGGACGAACAGGGCCACGG | sgRNA cloning in pJ4 |
| *SbTIL1* sg2 R | AAACCCGTGGCCCTGTTCGTCCTC | sgRNA cloning in pJ4 |
| *SbTIL1* sg3 F | AGCAGGGACCTGCCATGTAAGCAC | sgRNA cloning in pJ4 |
| *SbTIL1* sg3 R | AAACGTGCTTACATGGCAGGTCCC | sgRNA cloning in pJ4 |
| *SbTIL1* sg4 F | AGCAAAGGATATCTTCAGGGGCAA | sgRNA cloning in pJ4 |
| *SbTIL1* sg4 R | AAACTTGCCCCTGAAGATATCCTT | sgRNA cloning in pJ4 |
| *SbTIL1* sg5 F | AGCAAACGGCGGCAAGGTGCTCGT | sgRNA cloning in pJ4 |
| *SbTIL1* sg5 R | AAACACGAGCACCTTGCCGCCGTT | sgRNA cloning in pJ4 |
| *SbFT1* sg1 IVT F | GAAATTAATACGACTCACTATAGCGAGCTCAAGCCGTCCATGGGTTTTAGAGCTAGAAATAGCAAG | *In vitro* transcription |
| *SbFT1* sg2 IVT F | GAAATTAATACGACTCACTATAGGCCCAAGCGACCCAAATCTTGTTTTAGAGCTAGAAATAGCAAG | *In vitro* transcription |
| *SbFT8* sg2 IVT F | GAAATTAATACGACTCACTATAGTGTTGGGGGCACCGACCTCAGTTTTAGAGCTAGAAATAGCAAG | *In vitro* transcription |
| *SbFT8* sg4 IVT F | GAAATTAATACGACTCACTATAGCCTCAGGGTGTTCTATACACGTTTTAGAGCTAGAAATAGCAAG | *In vitro* transcription |
| *SbFT12* sg2 IVT F | GAAATTAATACGACTCACTATAGGCAAGTACTCCCTCAAGGTTGTTTTAGAGCTAGAAATAGCAAG | *In vitro* transcription |
| *SbFT12* sg5 IVT F | GAAATTAATACGACTCACTATAGCCCTCAAGGTTGGGTTGCTTGTTTTAGAGCTAGAAATAGCAAG | *In vitro* transcription |
| *SbTIL1* sg2 IVT F | GAAATTAATACGACTCACTATAGGAGGACGAACAGGGCCACGGGTTTTAGAGCTAGAAATAGCAAG | *In vitro* transcription |
| *SbTIL1* sg4 IVT F | GAAATTAATACGACTCACTATAGAACGGCGGCAAGGTGCTCGTGTTTTAGAGCTAGAAATAGCAAG | *In vitro* transcription |
| IVT universal R | AAAAAAGCACCGACTCGGTGCCACTTTTTCAAGTTGATAACGGACTAGCCTTATTTTAACTTGCTATTTCTAGCTCTAAAAC | *In vitro* transcription |
| *SbFT1* nested PCR F | CAGGGAAAGGGAGACGCT | Targeted deep sequencing |
| *SbFT1* nested PCR R | AGTGGTTCCTGGAATATCCGT | Targeted deep sequencing |
| *SbFT1* sg1, sg3, sg4 2nd PCR F | ACACTCTTTCCCTACACGACGCTCTTCCGATCTGTGCTGGACCCCTTCGTC | Targeted deep sequencing |
| *SbFT1* sg1, sg3, sg4 2nd PCR R | GACTGGAGTTCAGACGTGTGCTCTTCCGATCTCGTGACGAGCTAGCTAGAGA | Targeted deep sequencing |
| *SbFT1* sg2, sg5 2nd PCR F | ACACTCTTTCCCTACACGACGCTCTTCCGATCTAGTCTGTTGAGCTCGTACGT | Targeted deep sequencing |
| *SbFT1* sg2, sg5 2nd PCR R | GACTGGAGTTCAGACGTGTGCTCTTCCGATCTACATGCTACACAATGGACTAACA | Targeted deep sequencing |
| *SbFT8* nested PCR F | GACTGATGTGGAACCGTTGG | Targeted deep sequencing |
| *SbFT8* nested PCR R | TTGGCTCCAGTTGTTCCTG | Targeted deep sequencing |
| *SbFT8* 2nd PCR F | ACACTCTTTCCCTACACGACGCTCTTCCGATCTTGTGATACGTGATGTGTTGGA | Targeted deep sequencing |
| *SbFT8* 2nd PCR R | GACTGGAGTTCAGACGTGTGCTCTTCCGATCTTCCACATGACAGAGGCACAT | Targeted deep sequencing |
| *SbFT12* nested PCR F | GGCTAACGATTCCTTGGTTACT | Targeted deep sequencing |
| *SbFT12* nested PCR R | TCAGTTGATGCTGGGATGTC | Targeted deep sequencing |
| *SbFT12* sg1, sg3, sg4 2nd PCR F | ACACTCTTTCCCTACACGACGCTCTTCCGATCTCGTTGACATGATGATCCTATTCG | Targeted deep sequencing |
| *SbFT12* sg1, sg3, sg4 2nd PCR R | GACTGGAGTTCAGACGTGTGCTCTTCCGATCTATCCACGATACCACCAGCAT | Targeted deep sequencing |
| *SbFT12* sg2, sg5 2nd PCR F | ACACTCTTTCCCTACACGACGCTCTTCCGATCTTTAATTGATCGCAACACCATTTG | Targeted deep sequencing |
| *SbFT12* sg2, sg5 2nd PCR R | GACTGGAGTTCAGACGTGTGCTCTTCCGATCTCACCAAAATTTTATGGAGAAGCA | Targeted deep sequencing |
| *SbTIL1* nested PCR F | CACAAAGAGCGAGCAGAGAC | Targeted deep sequencing |
| *SbTIL1* nested PCR R | GTCGTCGTTCATCTTGGACA | Targeted deep sequencing |
| *SbTIL1* sg1, sg3 2nd PCR F | ACACTCTTTCCCTACACGACGCTCTTCCGATCTCTTCCGCTTCCACCCGAC | Targeted deep sequencing |
| *SbTIL1* sg1, sg3 2nd PCR R | GACTGGAGTTCAGACGTGTGCTCTTCCGATCTCCATGCATGATTCGACTCGT | Targeted deep sequencing |
| *SbTIL1* sg2, sg4, sg5 2nd PCR F | ACACTCTTTCCCTACACGACGCTCTTCCGATCTGGGGAGAAGGAGTGGTACTT | Targeted deep sequencing |
| *SbTIL1* sg2, sg4, sg5 2nd PCR R | GACTGGAGTTCAGACGTGTGCTCTTCCGATCTGGACTTCTCGCCCTTGGG | Targeted deep sequencing |
| *SbFT12* off-target nested PCR F | ATACACCGTCACACTGCAAC | Targeted deep sequencing |
| *SbFT12* off-target nested PCR R | GCACAGCTGTATGTACGTGG | Targeted deep sequencing |
| *SbFT12* off-target sg1, sg4 2nd PCR F | ACACTCTTTCCCTACACGACGCTCTTCCGATCTGAGATGTCCTGGACCCCT | Targeted deep sequencing |
| *SbFT12* off-target sg1, sg4 2nd PCR R | GACTGGAGTTCAGACGTGTGCTCTTCCGATCTGGCGACGACCAGCTATAG | Targeted deep sequencing |
| *SbFT12* off-target sg5 2nd PCR F | ACACTCTTTCCCTACACGACGCTCTTCCGATCTGTCTGATTATTTAACATGCATGGA | Targeted deep sequencing |
| *SbFT12* off-target sg5 2nd PCR R | GACTGGAGTTCAGACGTGTGCTCTTCCGATCTATTAGCTCTCTCATCATTGTCAAAA | Targeted deep sequencing |
