# Supplementary material for: A streamlined guide RNA screening system for genome editing in Sorghum bicolor
Source: Plant Methods. 2023 Aug 26;19:90. doi: 10.1186/s13007-023-01058-2 (PMC10463630; doi:10.1186/s13007-023-01058-2)
Supplement: Supplementary file 2 — Additional file 2: Sanger sequencing of relevant regions in target genes. a SbFT1, b SbFT8, c SbFT12, and d SbTIL1. Single nucleotide polymorphisms are indicated in turquoise. [file 13007_2023_1058_MOESM2_ESM.docx]

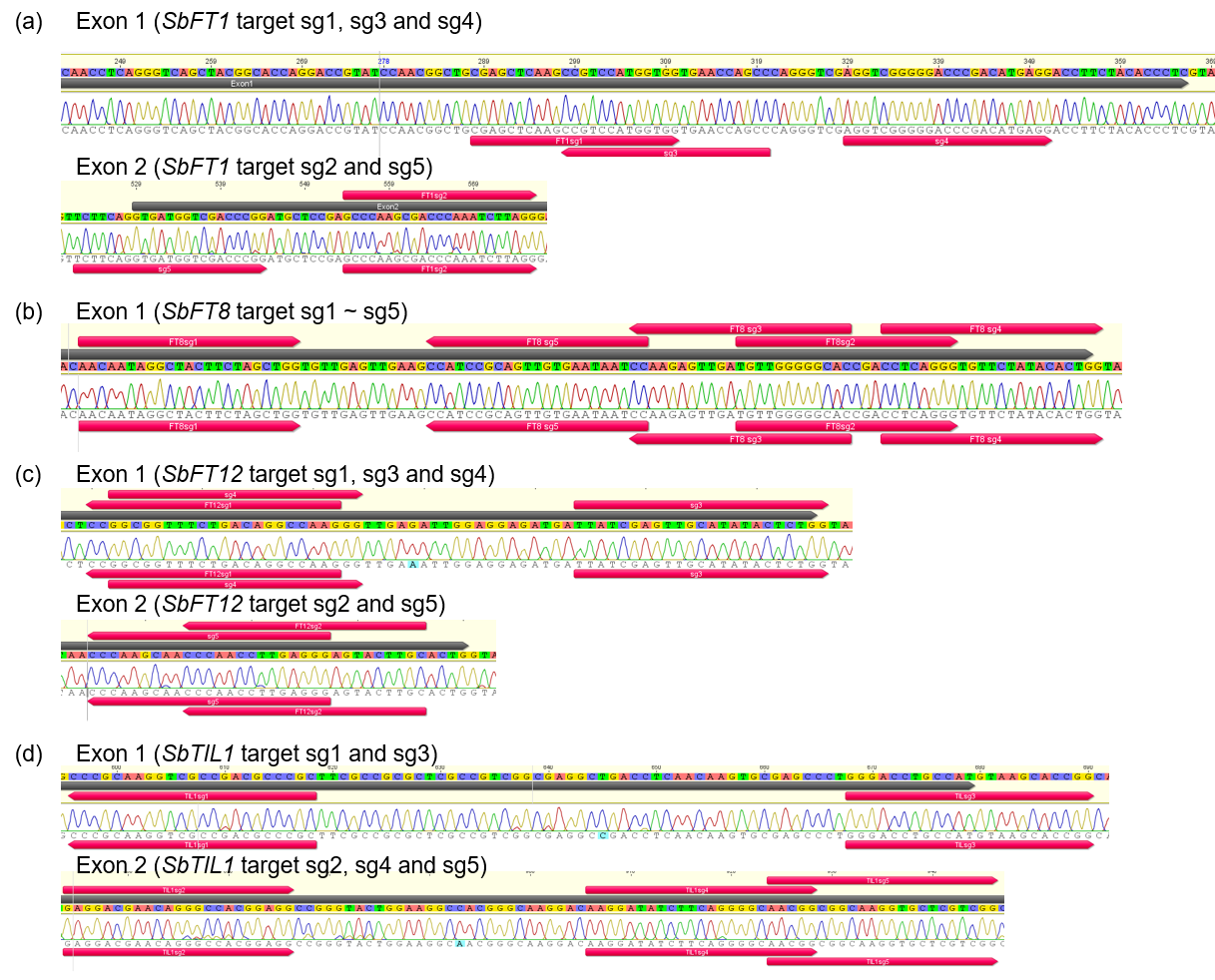


**Additional file 2.** Sanger sequencing of relevant regions in target genes. a: *SbFT1*, b: *SbFT8*, c: *SbFT12*, and d: *SbTIL1*. Single nucleotide polymorphisms are indicated in turquoise.
