# Supplementary material for: A streamlined guide RNA screening system for genome editing in Sorghum bicolor
Source: Plant Methods. 2023 Aug 26;19:90. doi: 10.1186/s13007-023-01058-2 (PMC10463630; doi:10.1186/s13007-023-01058-2)
Supplement: Supplementary file 3 — Additional file 3: Insertion of sgRNA-encoding sequences into the pJ4 plasmid. Target sequence annealed oligonucleotides were ligated into the BsaI-digested plasmid to construct vectors that express the desired sgRNAs. [file 13007_2023_1058_MOESM3_ESM.docx]

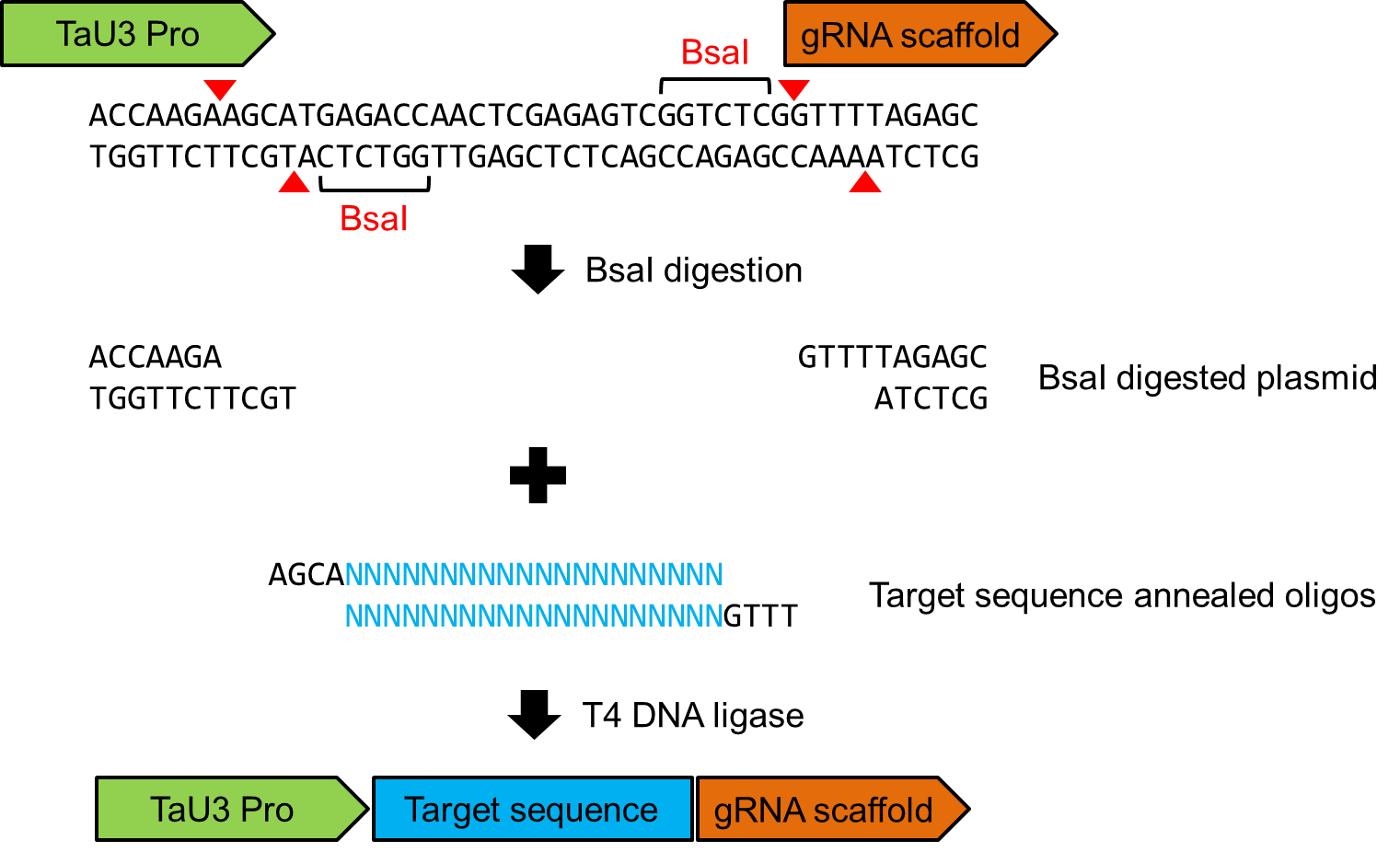


**Additional file 3.** Insertion of sgRNA-encoding sequences into the pJ4 plasmid. Target sequence annealed oligonucleotides were ligated into the BsaI-digested plasmid to construct vectors that express the desired sgRNAs.
