# Supplementary material for: A streamlined guide RNA screening system for genome editing in Sorghum bicolor
Source: Plant Methods. 2023 Aug 26;19:90. doi: 10.1186/s13007-023-01058-2 (PMC10463630; doi:10.1186/s13007-023-01058-2)
Supplement: Supplementary file 5 — Additional file 5: Indel patterns at endogenous sorghum loci induced following RNP-mediated delivery of CRISPR/Cas9 editing components. a SbFT1, b SbFT8, c SbFT12, and d SbTIL1. Total reads were obtained by targeted deep sequencing. PAM sequences and inserted or deleted nucleotides are indicated in blue and red, respectively. We tested n=3 biological replicates. [file 13007_2023_1058_MOESM5_ESM.docx]

a)


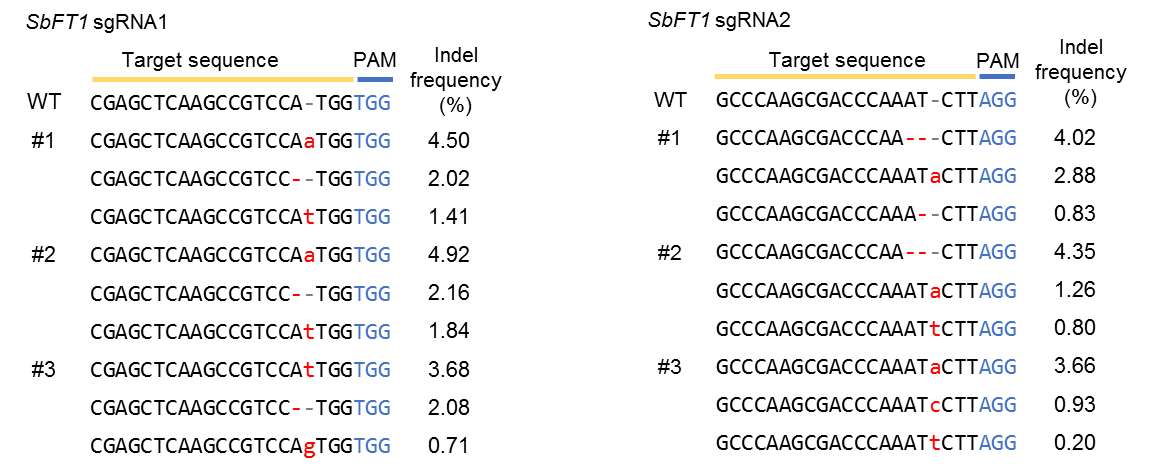


b)


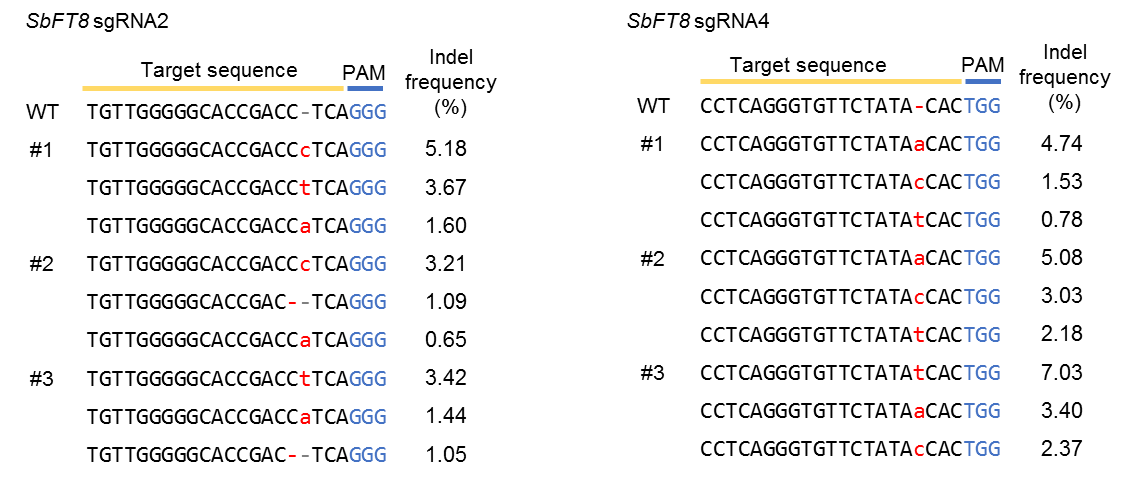


c)


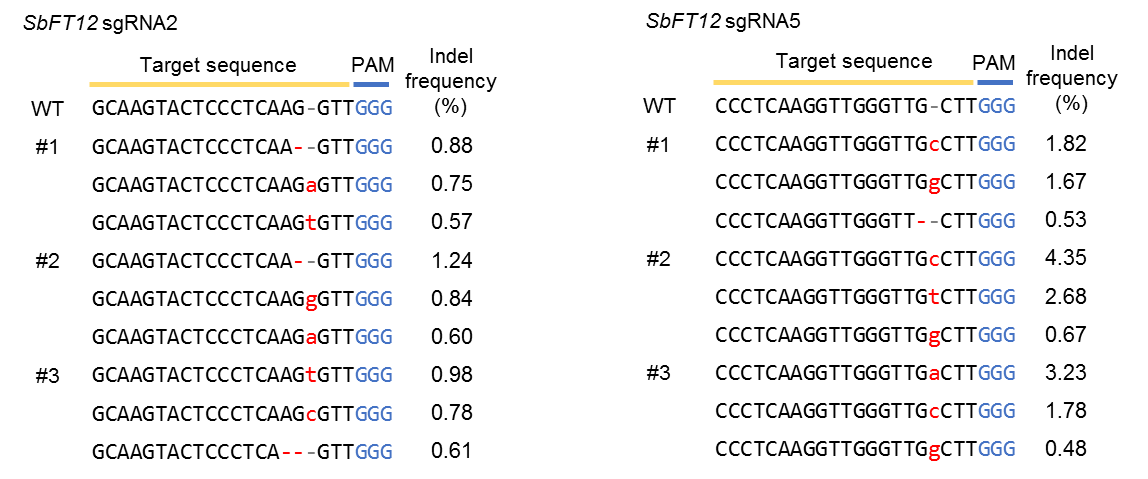


d)


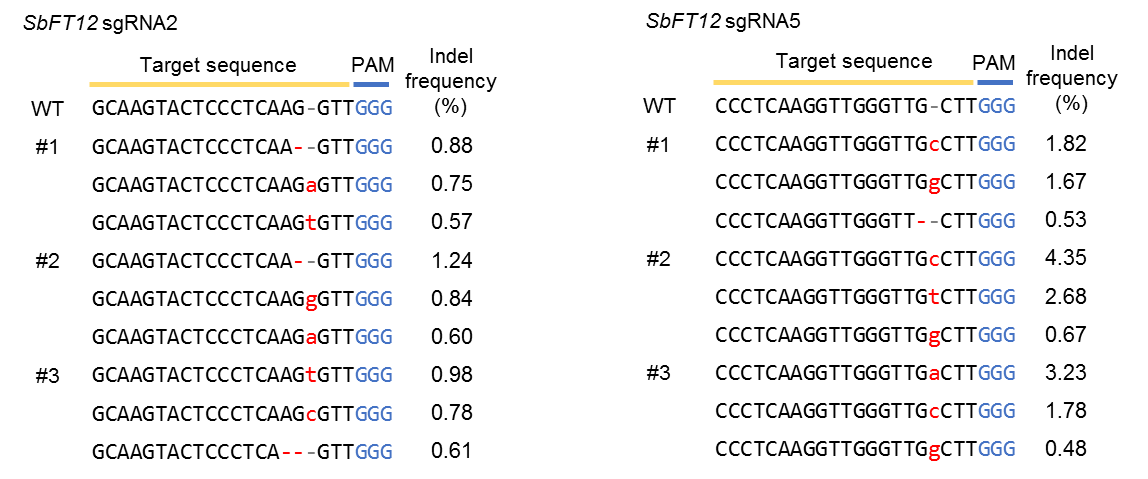


**Additional file 5.** Indel patterns at endogenous sorghum loci induced following RNP-mediated delivery of CRISPR/Cas9 editing components. a: *SbFT1*, b: *SbFT8*, c: *SbFT12*, and d: *SbTIL1*. Total reads were obtained by targeted deep sequencing. PAM sequences and inserted or deleted nucleotides are indicated in blue and red, respectively. We tested n=3 biological replicates.
